# The ratio of FoxA1 to FoxA2 in lung adenocarcinoma is regulated by LncRNA HOTAIR and chromatin remodeling factor LSH

**DOI:** 10.1038/srep17826

**Published:** 2015-12-11

**Authors:** Ranran Wang, Ying Shi, Ling Chen, Yiqun Jiang, Chao Mao, Bin Yan, Shuang Liu, Bin Shan, Yongguang Tao, Xiang Wang

**Affiliations:** 1 Department of Thoracic and Cardiovascular Surgery, Second Xiangya Hospital of Central South University, Changsha, China; 2 Cancer Research Institute, Central South University, Changsha, Hunan, 410078 China; 3 Key Laboratory of Carcinogenesis and Cancer Invasion, Ministry of Education, Hunan, 410078 China; 4 Key Laboratory of Carcinogenesis, Ministry of Health, Hunan, 410078 China; 5 Center for Medicine Research, Xiangya Hospital, Central South University, Changsha, Hunan, 410078 China; 6 College of Medical Sciences, Washington State University Spokane, 412 E. Spokane Falls Boulevard, Spokane, WA 99202, USA

## Abstract

The lncRNA HOTAIR is a critical regulator of cancer progression. Chromatin remodeling factor LSH is critical for normal development of plants and mammals. However, the underlying mechanisms causing this in cancer are not entirely clear. The functional diversification of the FOXA1 and FOXA2 contributes to the target genes during evolution and carcinogenesis. Little is known about the ratio of FOXA1 to FOXA2 in cancer. We here found that both HOTAIR and LSH overexpression was significantly correlated with poor survival in patients with lung adenocarcinoma cancer (ADC). Also, the ratio of FOXA1 and FOXA2 is linked with poor survival in patients with lung ADC. HOTAIR regulates the ratio of FOXA1 to FOXA2 and migration and invasion. HOTAIR and the ratio of FOXA1 to FOXA2 are negatively correlated. HOTAIR knockdown inhibits migration and invasion. HOTAIR is associated with LSH, and this association linked with the binding of LSH in the promoter of FOXA1, not FOXA2. Targeted inhibition of HOTAIR suppresses the migratory and invasive properties. These data suggest that HOTAIR is an important mediator of the ratio of FOXA1 and FOXA2 and LSH involves in, and suggest that HOTAIR inhibition may represent a promising therapeutic option for suppressing lung ADC progression.

Lung cancer is a leading cause of death worldwide, resulting in more than 1.3 million deaths per year, of which more than 40% are lung adenocarcinomas^1^. Furthermore, lung cancer is divided into small-cell lung cancer and non-small cell lung cancer (NSCLC) that includes adenocarcinoma (ADC) and squamous cell carcinoma (SCC), accounts for 80% to 85% of all lung cancer cases. Most often, tumors are discovered as locally advanced or metastatic disease, and despite improvements in molecular diagnosis and targeted therapies, the average 5 year survival rate for lung ADC is ∼15%^2^. The low survival rate is due to tumor recurrence and metastasis that is not sensitive to the traditional treatment. Thus, a detailed understanding of the mechanisms underlying NSCLC development and progression is essential for improving the diagnosis, prevention and therapy.

Long noncoding RNAs (lncRNAs) are defined as transcribed RNA molecules that are longer than 200 nucleotides and have no obvious protein coding capacity and are pervasively transcribed in mammalian genomes^3^. Human HOTAIR, a 2.2 kb RNA transcribed from the HOXC locus, binds both polycomb repressive complex 2 (PRC2) and LSD1 complexes and recruits them to hundreds of genomic sites to promote coordinated H3K27 methylation and H3K4 demethylation, respectively, for gene silencing^4^,^5^,^6^. HOTAIR silences human HOXD genes, a function that is believed to contribute to cell positional identity^6^, and overexpression of HOTAIR in several types of human cancers has been linked to metastasis, cancer progression and epithelial-to-mesenchymal transition^5^,^7^,^8^,^9^, indicating that HOTAIR functions as an oncogene. HOTAIR has been considered a prototype of lncRNA-guided chromatin modification that typifies a large class of lncRNAs associated with PRC2 and other chromatin modification complexes^10^. HOTAIR inactivation causes H3K4me3 gain and, to a lesser extent, H3K27me3 loss at Hox and additional genes^11^. Thus, function and target of HOTAIR in lung cancer remains unclear and is investigated in the current study.

LSH (lymphoid-specific helicase), also called HELLS (helicase, lymphoid specific) or PASG (proliferation-associated SNF2-like), a protein belonging to the SNF2 family of chromatin-remodeling ATPases, is critical for normal development of plants and mammals by establishing correct DNA methylation levels and patterns^12^,^13^,^14^,^15^. LSH serves as a target for DeltaNp63alpha driving skin tumorigenesis *in vivo* and co-operates with the oncogenic function of E2F3^16^,^17^. Interestingly, polycomb target genes are repressed by the histone H3 lysine 9 methytransferases G9a and GLP^18^. During lineage commitment and differentiation, LSH promotes binding of DNA methyltransferases and the G9a/GLP complex to specific loci and facilitates stable gene silencing via DNA methylation^15^. LSH is an important chromatin modifier in cancer where its function is unclear.

FOXA proteins belong to subclass A of the forkhead box containing transcription factor family^19^. Both FOXA1 and FOXA2 are essential for terminal differentiation and maturation of many endoderm-derived cells, including α-cells in the endocrine pancreas and liver, lung alveolar, and prostate luminal ductal epithelia^20^,^21^,^22^. Furthermore, FOXA1 and FOXA2 do not only cooperate in organogenesis, but also regulate target genes in a cell-type and stage-specific target binding^19^,^23^. However, while FOXA1 retains the more ancient role of regulating proliferation and growth by influencing DNA binding of p53, FOXA2 has acquired mutations in its DNA binding domain and a new role in regulating genes involved in lipid metabolism^24^. These findings suggest that the functional diversification of the FOXA1 and FOXA2 contributes to the target genes during evolution and carcinogenesis. Little is known about the role of FOXA1/2 in cancer even though their expression is observed in many human cancers including prostate, breast, liver, lung, and esophagus^19^,^23^. It is clear that FOXA family members play complementary roles in the regulation of organogenesis and gene expression^19^,^22^,^23^,^24^, indicating the ratio of FOXA1 to FOXA2 in a reasonable level is possibly involved in evolution and carcinogenesis. The involvement of ratio of FOXA1 to FOXA2 in lung cancer remains poorly known.

In this study, we investigated the expression pattern of HOTAIR in NSCLC tissues and analyzed its relationship to clinical pathological features. We also explored HOTAIR function relative to several other genes including FOXA1, FOXA2 and LSH. We found that HOTAIR formed an intact complex with LSH in turn, affected the ratio of FOXA1 to FOXA2 that could be regarded as a good biomarker for lung ADC.

## Results

### LncRNA HOTAIR expression levels increased in lung cancer and the ratio of FOXA1 to FOXA2 reversely correlated with HOTAIR

**Figure 1:**
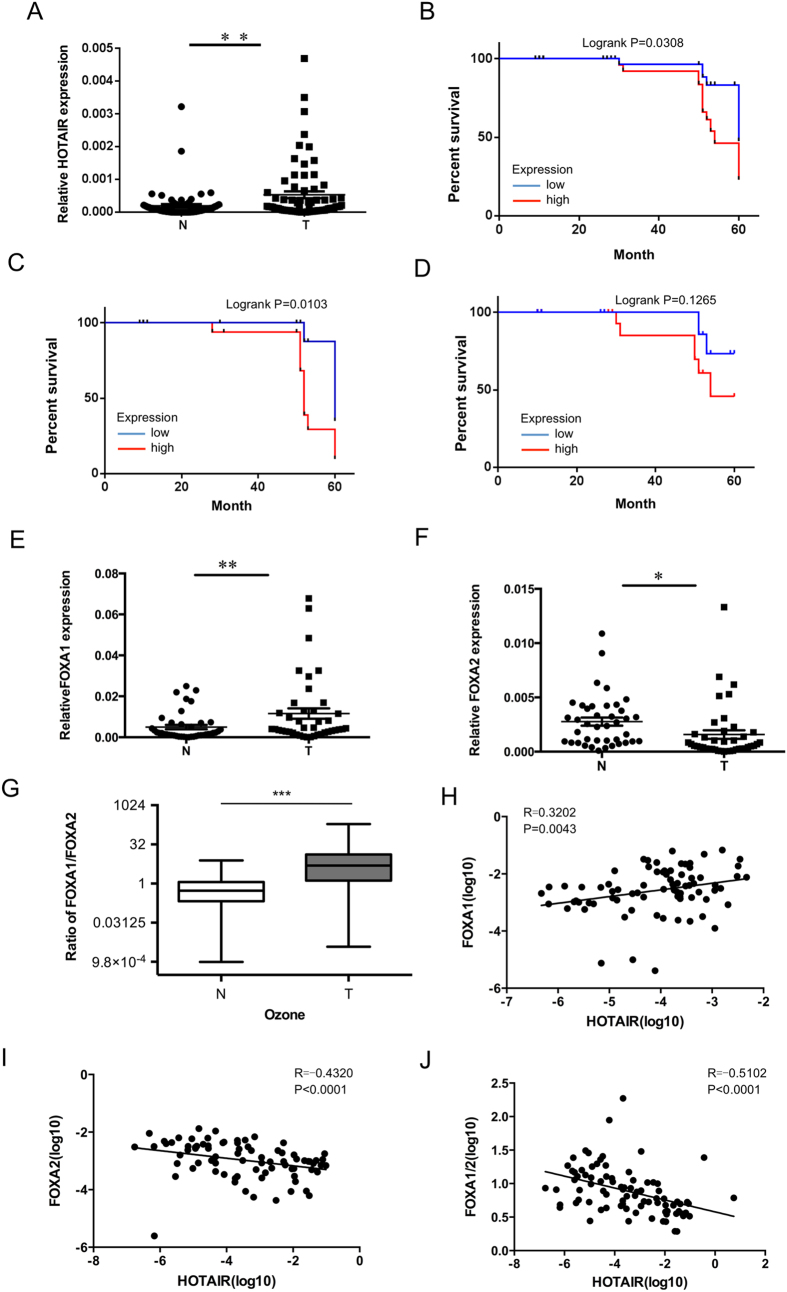
HOTAIR is upregulated in lung cancer and the ratio of FOXA1 to FOXA2 reversely correlates with HOTAIR. (**A**) Dot blot analysis, based on quantitative reverse transcription-polymerase chain reaction (qRT-PCR) analysis expression of HOTAIR in 73 paired lung cancer samples, and corresponding normal lung tissues. Kaplan-Meier curves for overall survival of the related samples measured in lung cancer (**B**), lung ADCs (**C**) and lung SCCs (**D**). Dot blot analysis expression of FOXA1 (**E**), FOXA2 (**F**) and the ratio of FOXA1 to FOXA2 (**G**) in lung cancer samples measured in (A). *P < 0.05. **P < 0.01. ***P < 0.001. The correlation of HOTAIR with FOXA1 (H), FOXA2 (**I**), and the ratio of FOXA1 to FOXA2 (**J**) were analyzed.

We detected the HOTAIR level in an independent panel of 73 primary lung tumors with extensive clinical follow-up, quantitative PCR showed that HOTAIR was overexpressed from hundreds to nearly two-thousand-fold in lung cancer metastases, and the HOTAIR expression level was sometimes high but heterogeneous among primary tumors (Fig. 1A). Multivariate analysis showed that the expression level of HOTAIR was independent of clinical risk factor such as gender, smoking, tumor differentiation and tumor size, but linked with clinical stage and lymphatic metastasis (Table 1). Of note, Kaplan-Meier plotter performed on a cohort of these lung cancers showed that lower expression of HOTAIR linked with overall survival in all lung cancer (Fig. 1B), and ADCs (Fig. 1C), but not in lung SCCs (Fig. 1D).

Next, we analyzed FOXA1 and FOXA2 levels in NSCLC and normal lung tissues. Fig. 1E showed that FOXA1 increased in lung caner and Fig. 1F demonstrated that FOXA2 decreased in lung cancer. Interestingly, the ratio of FoxA1/FoxA2 increased in lung cancer as compare with normal lung tissues (Fig. 1G). Furthermore, we found that FOXA1 expression level was positively related with HOTAIR level (Fig. 1H). Meanwhile, the expression of FOXA2 and the ratio of FOXA1/FOXA2 were reversely correlated with the expression level of HOTAIR (Fig. 1I,J), indicating that HOTAIR is possibly associated with FOXA1 and FOXA2.

### Overexpression of HOTAIR promoted migration and invasion in lung ADC cells

**Figure 2:**
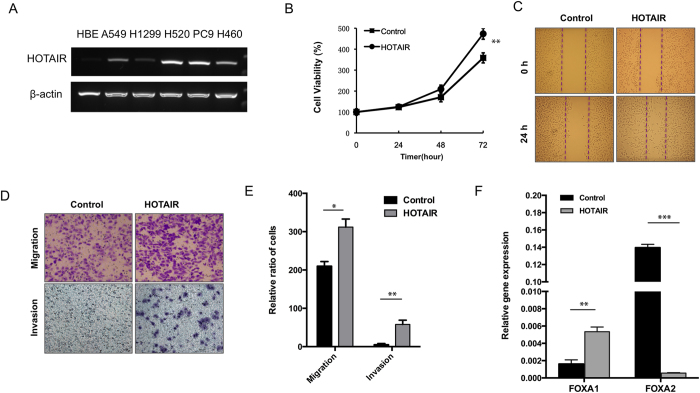
Overexpression of HOTAIR promotes migration and invasion in lung ADC cells. (**A**) HOTAIR expression level in several lung cancer cell lines. (**B**) Cell growth analysis was performed after stable expression of HOTAIR in A549 cells. (**C**) Wound-healing analysis were measured in A549 cells that stably overexpressed HOTAIR. (**D,E**) Migration and invasion were analyzed in A549 cells with ectopic expression of HOATIR. The means and s.d. values were derived from three independent experiments. *P < 0.05, **P < 0.01, ***P < 0.001. (**F**) qRT-PCR was analyzed FOXA1, FOXA2 in the presence of HOTAIR in A549 cells.

We next examined the effects of manipulating HOTAIR level in several lung cancer cell lines including lung ADC cells H1299, PC9 and A549, and lung SCC cells H520. HOTAIR levels were significantly higher in lung cancer cell lines than that in HBE (human bronchial epithelial cell) (Fig. 2A), A549 was selected for further study for it’s one of lowest levels of HOTAIR in these cancer cells. After stable ectopic expression of HOTAIR in A549 cells, we found that HOTAIR promoted cell growth as compare with the control group (Fig. 2B). Moreover, stable expression of HOTAIR in A549 cells resulted in increase of migration in a wound healing assay in 24 h (Fig. 2C). Stable expression of HOTAIR showed an increased activity to migration and invasion (Fig. 2D,E). Then, we detected the effect of HOTAIR on mRNA levels of FOXA1 and FOXA2, and we found that stable expression of HOTAIR increased mRNA levels FOXA1, whereas HOTAIR decreased FOXA2 mRNA level (Fig. 2F).

### Knockdown of HOTAIR reduced migration and invasion in lung ADC cells

**Figure 3:**
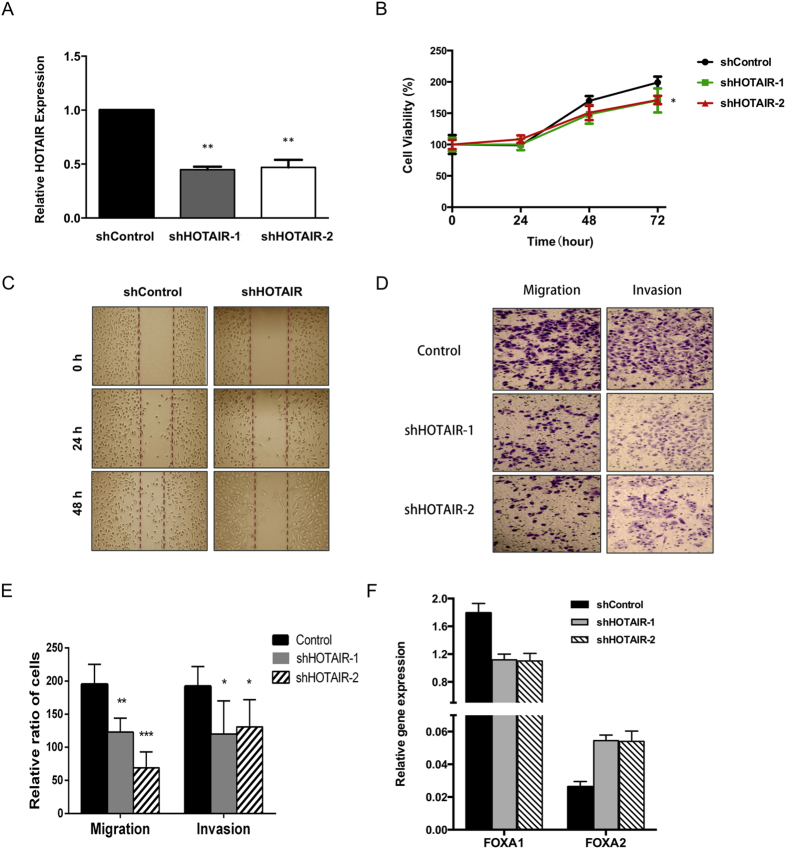
Knockdown of HOTAIR attenuates migration and invasion in lung ADC cells. (**A**) shRNA mediated knockdown of HOTAIR in PC9 cells. (**B**) Cell growth analysis was performed after stable knockdown of HOTAIR in PC9 cells. (**C**) Wound-healing analysis were measured in PC9 cells that stably knockdown of HOTAIR. (**D,E**) Migration and invasion were analyzed in PC9 cells in the depletion of HOATIR. The means and s.d. values were derived from three independent experiments. *P < 0.05, **P < 0.01, ***P < 0.001. (**F**) qRT-PCR was analyzed FOXA1, FOXA2 after knockdown of HOTAIR in PC9 cells.

To further understand the physiological role of HOTAIR in lung ADC cells, we generated stable HOTAIR knockdown in PC9 cancer cells using a set of shHOTAIR lentivirus vectors (see ‘Materials and Method’ section) and observed about 70% of reduction in HOTAIR levels (Fig. 3A). Stable knockdown of HOTAIR resulted in reduced growth of PC9 cells during culture (Fig. 3B). Stable knockdown of HOTAIR resulted in a decreased migration activity in wound healing assay (Fig. 3C). Furthermore, stable knockdown of HOTAIR in PC9 cells resulted in a decreased activity to migration and invasion (Fig. 3D,E).

Then, we detected the effect of HOTAIR on mRNA levels of FOXA1 and FOXA2 after the depletion of HOTAIR, we found that stable knockdown of HOTAIR increased mRNA levels of FOXA2, but depletion of HOTAIR decreased the expression level of FOXA1 significantly(Fig. 3F), clearly knockdown of HOHAIR increased the ratio of FOXA1 to FOXA2.

### HOTAIR interacted with chromatin remodeling factor LSH

**Figure 4:**
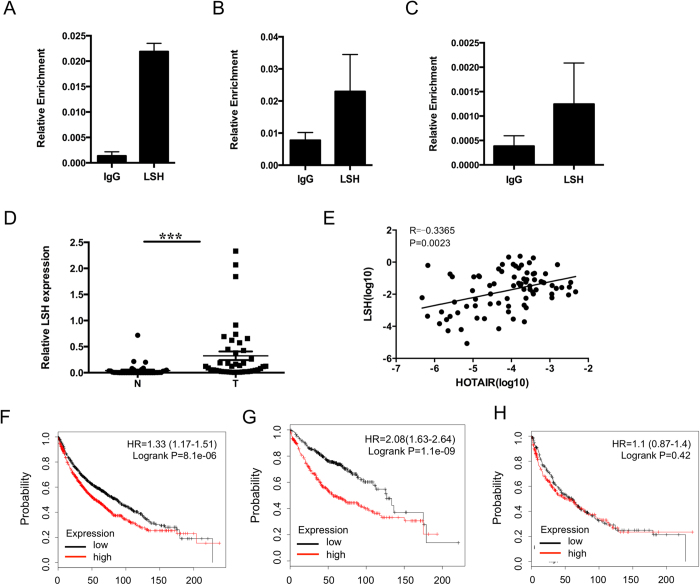
HOTAIR interacts with chromatin remodeling factor LSH. Immunoprecipitation of LSH retrieves HOTAIR. Nuclear extracts of PC9 (**A**), H1299 (**B**) and A549 with overexpression of HOTAIR (**C**) were immunoprecipiated by IgG, anti-LSH. Coprecipitated RNAs were detected by RT-PCR using primers for HOTAIR. (**D**) qRT-PCR analysis expression of LSH in 73 paired lung cancer samples, and corresponding normal lung tissues. (**E**) The correlation of HOTAIR with LSH was analyzed. An in silico meta-analysis of LSH expression profiles with Kaplan-Meier plotter in all lung cancers (**F**), ADCs (**G**) and SCCs (**H**).

HOTAIR binds both polycomb repressive complex 2 (PRC2) and LSH can associate with PRC1 components and influence PRC-mediated histone modifications^6^,^25^, indicating that a potential interaction of LSH and HOTAIR exists. To test this hypothesis, we performed RNA Immunoprecipitation (RIP) using anti-LSH antibody. Data in Fig. 4A showed that LSH interacted with HOTAIR in PC9, similar findings were shown in H1299 cells. Moreover, we found that LSH formed an intact complex of A549 after stable overexpression of HOTAIR, indicating that LSH interacted with HOTAIR.

Next, to address the role of LSH in lung cancer, we detected the LSH mRNA level in an independent panel of 60 primary lung tumors. We found that the mRNA level of LSH was highly expressed lung cancer tumors (Fig. 4D). Furthermore, we found that LSH expression level was positively related with HOTAIR level (Fig. 4E).

Lastly, to further address the role of LSH in lung cancers, an *in silico* meta-analysis of LSH expression profiles with Kaplan-Meier plotter (http://kmplot.com) was performed. A cohort of these lung cancers showed that lower expression of LSH at mRNA level linked with overall survival in all lung cancer (Fig. 4F) and lung ADCs (Fig. 4G), not lung SCCs (Fig. 4H).

### LSH directly regulated FOXA1 gene and positively linked with the ratio of FOXA1 to FOXA2

**Figure 5:**
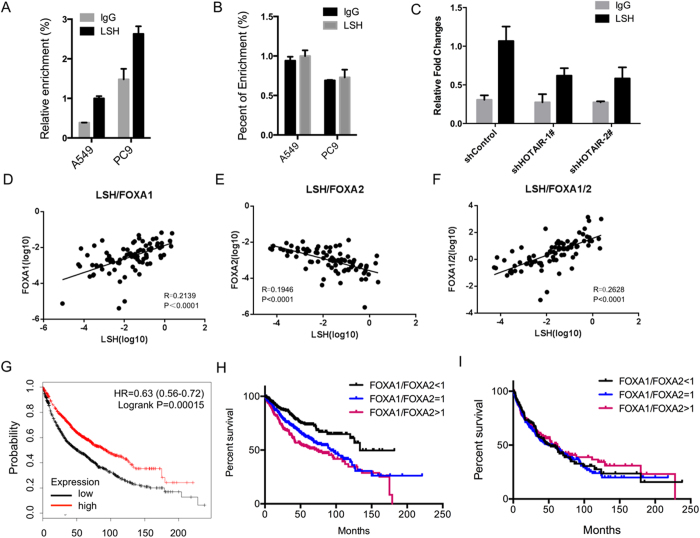
LSH regulates FOXA1 gene and links with the ratio of FOXA1 to FOXA2. ChIP analysis of A549 and PC9 cells were performed to detect LSH around the transcriptional start sites in the FOXA1 (**A**) and FOXA2 (**B**) promoters. (**C**) ChIP analysis was performed to detect LSH around the transcriptional start sites in FOXA1 promoter after stable knockdown of HOTAIR in PC9 cells. The correlation of LSH with FOXA1 (**D**), FOXA2 (**E**), and the ratio of FOXA1 to FOXA2 (**F**) were analyzed. An in silico meta-analysis of FOXA1 expression profiles with Kaplan-Meier plotter in all lung cancers (**G**). An in silico meta-analysis of the ration of FOXA1 to FOXA2 with Kaplan-Meier plotter in ADCs (**H**) and SCCs (**I**).

To address whether LSH could bind to the promoters of FOXA1 and FOXA2, we performed ChIP assay in A549 and PC9 cells using anti-LSH antibody. Data in Fig. 5A indicated that LSH could bind to the promoter of FOXA1, while LSH did not bind to the promoter of FOXA2 at all (Fig. 5B). Furthermore, stable knockdown of HOTAIR in PC9 cells resulted in a decreased binding of LSH to the FOXA1 promoter, indicating that LSH functions as activator in the regulation of FOXA1 in a dependent manner of HOTAIR.

Next, we found that FOXA1 expression level was positively related with LSH level (Fig. 5D). Meanwhile, the expression of FOXA2 was negatively linked with LSH level (Fig. 5E). Moreover, the ratio of FOXA1/FOXA2 was correlated with the expression level of LSH (Fig. 5F).

Lastly, to further understand the role of FOXA1 and FOXA2 in lung cancers, we performed an *in silico* meta-analysis of FOXA1 and FOXA2 expression profiles with Kaplan-Meier plotter (http://kmplot.com). Interestingly, a cohort of these lung cancers showed that only higher expression of FOXA1, not FOXA2, linked with overall survival in all lung cancer (Fig. 5F), but not in lung ADCs and SCCs (data not shown). Moreover, we found that the ratio of FOXA1 to FOXA2 linked with overall survival in lung ADCs (Fig. 5H), not in lung SCCs (Fig. 5I).

## Discussion

In this study, we showed that the expression pattern of HOTAIR in lung cancer tissues and analyzed its relationship to clinical pathological features. We also explored HOTAIR function relative to several other genes including FOXA1, FOXA2 and LSH. Furthermore, we found that LSH interacted with HOTAIR, in turn, the intact complex affects the ratio of FOXA1 to FOXA2, in a dependent manner, which could be regarded as a good biomarker for lung ADCs.

HOTAIR, a widely focused LncRNA, was initially proposed to be involved in primary breast cancer and breast cancer metastasis, numerous next studies have clearly demonstrated the importance of HOTAIR in tumors, immerging as a promising diagnostic and therapeutic target for several malignancy including lung cancer^5^,^7^,^26^,^27^,^28^,^29^,^30^,^31^,^32^. Here, we provide evidence that HOTAIR was closely linked with lung ADCs, not SCCs, including clinical stage and lymphatic metastasis. HOTAIR binds to polycomb repressive complex 2 (PRC2) and LSD1/CoREST/REST complex, to repress the expression of the homeobox gene D cluster (HOXD)^5^,^33^. PRC2 contains Enhancer of Zeste Homolog 2 (EZH2), a histone methyltransferase that marks a gene for transcriptional repression via trimethylation of histone H3 Lys27 (H3K27me3)^34^, and the LSD1/CoREST/REST complex contains Lysine-Specific Demethylase 1(LSD1), a histone demethylase that inactivates gene expression via demethylation of the dimethylated histone H3 Lys4 (H3K4me2), a histone modification that is critical for transcriptional activation^35^. PRC2 and LSD1/CoREST/REST complex play important roles in the epigenetic regulation of gene expression, it is not surprising that HOTAIR is deregulated so many types of cancer. Here, we provide evidence in the interaction between HOTAIR and LSH, a novel protein partner.

LSH (lymphoid-specific helicase), also called HELLS (helicase, lymphoid specific) or PASG (proliferation-associated SNF2-like), a protein belonging to the SNF2 family of chromatin-remodeling ATPases, is critical for normal development of plants and mammals by establishing correct DNA methylation levels and patterns^12^,^13^,^14^,^15^. LSH maintains genome stability in mammalian somatic cells^36^,^37^. LSH serves as a target for DeltaNp63alpha driving skin tumorigenesis *in vivo* and co-operates with the oncogenic function of E2F3^16^,^17^. Reports show that LSH contributes to the malignant progression of prostate cancer, melanoma, and head and neck cancer, etc^17^,^38^,^39^. Loss of function of LSH (HELLS) by allelic loss and aberrant proteins by tumor-specific exon creation may result in epigenetic deregulation, leading lung cells to malignancy or its progression^40^. However, the exactly role of LSH in lung cancer remains unclear. Here, we found that the interaction of LSH with HOTAIR might involve in lung cancer and LSH might bind to the target gene directly as a chromatin modifier. Up to now, it is the first report that LSH displays a novel epigenetic function by interaction of LncRNA beyond DNA methylation.

FOXA1 and FOXA2 oppositely regulate target gene, indicating that a balance between FOXA1 and FOXA2 during organ development^19^. Furthermore, FOXA1 and FOXA2 have unique targets in addition to many common ones, indicative of diverged function^24^. Here, we provide evidence that LSH bound to the promoter of FOXA1, not FOXA2, then increased the ratio of FOXA1 and FOXA2 at mRNA level. Knockdown of FOXA1 and FOXA2 results in increased cell migration in pancreatic cancer respectively^41^, indicating that both FOXA1 and FOXA2 inhibit transition of the epithelial stage to mesenchymal stage as a tumor suppressor, however, another reports FOXA1 functions a tumor promoter^42^ and FOXA2 is regarded as a tumor suppressor^43^,^44^. These indicate that the functions of FOXA1 and FOXA2 are complex. Here, we show that both LSH and HOTAIR are positively related with FOXA1, but not FOXA2, in lung ADCs. Moreover, the ratio of FOXA1 and FOXA2 is affected by LSH and is closely linked lung ADCs, indicating the ratio of FOXA1 and FOXA2 is a good biomarker in lung ADCs. The oncogenic functions of HOTAIR may be partially exerted through its affect on the epithelial-mesenchymal transition; however, further experiments are needed to elucidate the precise molecular mechanisms by which HOTAIR regulates FOXA1/2.

These data demonstrate that HOTAIR could be an important biochemical index of the patients in lung ADCs and it may serves as a potential marker in ADC survival. The interaction of HOTAIR with LSH might be emerged as a novel master regulator of lung ADC. Moreover, The ratio of FOXA1 to FOXA2 is regarded as a good biomarker for lung ADC under the regulation of both HOTAIR.

## Methods

### Tissue samples

Seventy-three paired NSCLC and adjacent non-tumor lung tissues were obtained from patients who underwent surgery at the second hospital of Xiangya (Hunan, China) during 2010–2014 and were diagnosed with NSCLC (stage I, II, and III) based on histopathological evaluation. Clinical characteristics including tumor-node-metastasis (TNM) stage were collected. No local or systemic treatment was conducted in these patients before surgery. All collected tissue samples were immediately snap-frozen in liquid nitrogen. The Research Ethics Committee of the second Xiangya hospital approved the study. The study is reported in accordance with the approved guidelines, Written informed consent was obtained from all patients.

### Cell culture

Three human NSCLC adenocarcinoma cell lines (A549, PC9, H1299), one NSCLC squamous carcinomas cell line (H520), and one normal human bronchial epithelial cell line (HBE) were purchased from the ATCC or Cell Biology of the Chinese Academy of Sciences (Shanghai, China). Cells were cultured in RPMI 1640 or DMEM (GIBCO-BRL) medium supplemented with 10% fetal bovine serum (FBS), 100 U/ml penicillin and 100 mg/ml streptomycin (Invitrogen, Carlsbad, CA, USA) in humidified air with 5% CO_2_ at 37 °C.

### HOTAIR expression and shRNA constructs and Cell transfection

The HOTAIR expression construct LZRS-HOTAIR was kindly provided by dr. Howard Chang, Stanford University, USA, and the HOTAIR coding region was subcloned into the retroviral vector pLVX-EF1α-IRES-Puro (Clontech). HOTAIR shRNA vectors (GV248, HOTAIR-shRNA 1, 2, 3 and control shRNA) were purchased from GeneChem (Shanghai, China). Transfection of plasmids was performed using Lipofectamine 2000 (Invitrogen, Carlsbad, CA) according to the manufacturer’s protocol. Stable shRNA expressing colonies were selected using puromycin.

### RNA extraction and quantitative real-time PCR

Total RNA was extracted from tissues or cultured cells with TRIzol reagent (Invitrogen), and the reverse transcription reactions were performed using random primers. Quantitative real-time PCR was performed using SYBR Premix Ex Taq (TaKaRa, Dalian, China) on ABI7500 Fast Real-Time PCR System (Applied Biosystems). A dissociation step was performed to generate a melting curve to confirm the specificity of the amplification. β-actin was used as the reference gene. The relative levels of gene expression were represented as ΔCt = Ct gene-Ct reference, and the fold changes in gene expression were calculated by the 2^−ΔΔCt^ method. The primer sequences were provided in the following, HOTAIR forward primer: 5′- GCAGTGGAATGGAACGGATT-3′; reverse primer, 5′- CGTGGCATTTCTGGTCTTGTA-3′. FOXA1 forward primer: 5′-GCAATACTCGCCTTACGGCT-3′, reverse primer: 5′-TACACACCTTGGTAGTACGCC-3′; FOXA2 forward primer: 5′-GGAGCAGCTACTATGCAGAGC-3′; reverse primer: 5′-CGTGTTCATGCCGTTCATCC-3′; LSH forward primer: 5′-AGAAGGCATGGAATGGCTTAGG-3′, reverse primer: 5′-GCCACAGACAAGAAAAGGTCC-3′; β-actin forward primer: 5′-GCACCACACCTTCTACAATGAG -3′, reverse primer: 5′-GATAGCACAGCCTGGATAGCA -3′. Experiments were repeated in triplicate.

### Cell proliferation assays

Cell proliferation was monitored using Cell Proliferation Reagent Kit I (MTS). Over expression HOTAIR Si-HOTAIR-transfected PC9 cells (5000/well) were cultured in 96-well plates. Cell proliferation was documented every 24h following the manufacturer’s protocol. All experiments were performed in quadruplicate.

### Wound-Healing Assay

A confluent monolayer of lung cancer cells (1 × 10^6^ cells) was cultured overnight and a scratch was introduced with a pipette tip. Then images were captured at time as indicated using a light microscope.

### Cell migration and invasion assays

For the migration assays, 48 h after transfection, 5 × 10^4^ cells in serum-free media were placed into the upper chamber of an insert (8 μm pore size, Corning). For invasion assays, 2 × 10^5^ cells in serum-free medium were placed into the upper chamber of an insert coated with Matrigel (BD Biosciences, Sanose, CA). Media containing 10% FBS were added to the lower chamber. After incubation for 24 hours, the cells remaining on the upper membrane were removed with cotton wool, whereas the cells that had migrated or invaded through the membrane were stained with methanol and 0.1% crystal violet, imaged, and counted using inverted microscope (Olympus, Tokyo, Japan). Experiments were independently repeated three times.

### RNA immunoprecipitation

RNA immunoprecipitation (RIP) or RNA pulldown was performed as described previously^6^. RIP experiments were carried out using a Magna RIP RNA-Binding Protein Immunoprecipitation Kit (Millipore) according to the manufacturer’s instructions. Antibody for RIP assays of LSH (Sigma) was diluted as 1:1,000. The coprecipitated RNAs were detected by reverse transcription PCR. The primes for detecting malat1 were listed in HOTAIR forward primer: 5′- GCAGTGGAATGGAACGGATT-3′; reverse primer, 5′- CGTGGCATTTCTGGTCTTGTA-3′. Experiments were independently repeated three times.

### Chromatin immunoprecipitation

Chromatin immunoprecipitation (ChIP) was carried out as described previously^45^,^46^. Briefly, cross-linked chromatin was sonicated into 200- to 1,000-bp fragments. Then, the chromatin was immunoprecipitated using anti-LSH antibody. Quantitative PCR was conducted according to the method described above. Primers are listed in the following: FOXA1 forward primer: 5′- TTGTAGGTGCGAGCGTCTT-3′; reverse primer: 5′-AAGTGCAGTTGAGCTGATGTG-3′; FOXA2 forward primer: 5′- CACCTCCTGCCCTGTTTGTT-3′, reverse primer: 5′- GCTCTCCGACTCCTCAGACA-3′

### Statistical analysis and an *in silico* meta-analysis

Student’s t-test (2-tailed), one-way ANOVA, and Spearman correlation analysis using SPSS 22.0 software (IBM, IL, USA). *P*-values of less than 0.05 were considered significant. An *in silico* meta-analysis of IKKα expression profiles with Kaplan-Meier plotter from the webstie (https://kmplot.com).

## Additional Information

**How to cite this article**: Wang, R. *et al.* The ratio of FoxA1 to FoxA2 in lung adenocarcinoma is regulated by LncRNA HOTAIR and chromatin remodeling factor LSH. *Sci. Rep.* **5**, 17826; doi: 10.1038/srep17826 (2015).

## Figures and Tables

**Table 1:** HOTAIR expression level and clinical characteristics of lung cancer patients

**Factors**	***n*****(%)**	**Relative expression level (Mean)**	***P*** **value**
Gender			
Male	52 (71)	1.81	0.164
Female	21 (29)	1.06	
Smoking History			
Yes	38 (52)	1.88	0.358
No	35 (48)	1.17	
Histology			
ADC	32 (44)	1.74	0.282
SCC	41 (56)	1.46	
Differentiation			
Poor and moderate	67 (92)	1.64	0.441
Well	6 (8)	1.87	
T stage			
T1	15 (20)	0.93	0.390
T2	56 (77)	1.65	
T3	2 (3)	2.68	
N stage			
N0	41 (56)	0.90	0.026
N1-N3	32 (44)	2.38	
Clinical Stages			
I-II	55 (75)	1.24	0.005
III-IV	18 (25)	3.01	
